# Impact of maintenance immunosuppressive therapy on the fecal microbiome of renal transplant recipients: Comparison between an everolimus- and a standard tacrolimus-based regimen

**DOI:** 10.1371/journal.pone.0178228

**Published:** 2017-05-24

**Authors:** Gianluigi Zaza, Alessandra Dalla Gassa, Giovanna Felis, Simona Granata, Sandra Torriani, Antonio Lupo

**Affiliations:** 1 Renal Unit, Department of Medicine, University Hospital of Verona, Verona, Italy; 2 Department of Biotechnology, University of Verona, Verona, Italy; University of Toledo, UNITED STATES

## Abstract

**Background:**

The gut microbiome is the full set of microbes living in the gastrointestinal tract and is emerging as an important dynamic/fluid system that, if altered by environmental, dietetic or pharmacological factors, could considerably influence drug response. However, the immunosuppressive drug-induced modifications of this system are still poorly defined.

**Methods:**

We employed an innovative bioinformatics approach to assess differences in the whole-gut microbial metagenomic profile of 20 renal transplant recipients undergoing maintenance treatment with two different immunosuppressive protocols. Nine patients were treated with everolimus plus mycophenolate mofetil (EVE+MMF group), and 11 patients were treated with a standard therapy with tacrolimus plus mycophenolate mofetil (TAC+MMF group).

**Results:**

A statistical analysis of comparative high-throughput data demonstrated that although similar according to the degree of Shannon diversity (alpha diversity) at the taxonomic level, three functional genes clearly discriminated EVE+MMF *versus* TAC+MMF (cutoff: log2 fold change≥1, FDR≤0.05). Flagellar motor switch protein (fliNY) and type IV pilus assembly protein pilM (pilM) were significantly enriched in TAC+MMF-treated patients, while macrolide transport system mrsA (msrA) was more abundant in patients treated with EVE+MMF. Finally, PERMANOVA revealed that among the variables analyzed and included in our model, only the consumption of sugar significantly influenced beta diversity.

**Conclusions:**

Our study, although performed on a relatively small number of patients, showed, for the first time, specific immunosuppressive-related effects on fecal microbiome of renal transplant recipients and it suggested that the analysis of the gut microbes community could represent a new tool to better understand the effects of drugs currently employed in organ transplantations. However, multicenter studies including healthy controls should be undertaken to better address this objective.

## Introduction

In recent years, advances in biotechnology have increased our knowledge of the human intestinal microbiome, the entire collection of the genomic elements of a specific microbiota [1]. In particular, metagenomics, through the sequencing of bacterial genomic DNA from the gut, has permitted the evaluation of the genetic potential and complexity of the microbial population from the gastrointestinal tract in several clinical settings. Additionally, recent studies have evaluated the role of the gut microbiome in the drug response and the possible impact of the drugs on the composition of the gut microbiota with clinical, metabolic/biochemical and immunological consequences [2].

However, although the study of the gut microbiome is a recent emerging field in medicine, few reports have measured the modification of this complex system in renal transplant recipients [3,4]. Furthermore, no analyses have been conducted to deeply analyze genomic changes induced by specific immunosuppressive medications (particularly mTOR-inhibitors) chronically employed in this population to avoid a rapid loss of graft function.

mTOR-inhibitors (mTOR-I) are less frequently employed immunosuppressive medications that act by inhibiting the mammalian target of rapamycin (mTOR), a regulatory protein kinase primarily involved in several biological functions (e.g., protein synthesis, autophagy, cytoskeleton remodeling) essential for lymphocyte proliferation.

The more commonly used calcineurin inhibitors (cyclosporine and tacrolimus) suppress the immune system by preventing interleukin-2 production in T cells. Both drug categories can be used alone, combined or co-administered with glucocorticoids (methylprednisolone, prednisolone) and antiproliferative agents (azathioprine, mycophenolate mofetil).

Based on available data obtained by studies performed in different fields of medicine, it is plausible that the continuous administration of immunosuppressive drugs in transplanted patients may alter their intestinal microbial balance with a potential clinical impact and a central role in the onset and development of systemic complications (e.g., recurrent infections, reduced response to antibiotics, metabolic and cardiovascular alterations). Additionally, the modification of the intestinal microbe-associated functional integrity of the gut microbiota may contribute to the correct pharmacokinetics of these drugs, causing important, severe adverse renal and systemic effects.

Recently, renal transplant recipients who developed post-transplant diarrhea, a frequent complication with a significant clinical impact on graft survival, were shown to possess reduced saccharolytic bacteria (e.g., *Dorea*, *Coprococcus*, *Ruminococcus*, *Bacteroides*) commonly associated with a state of intestinal homeostasis. The authors hypothesized that the use of probiotics including these species could represent a complementary strategy to avoid this complication. The study further described a reduction in *Bacteroides* and an increase in *Proteobacteria* in transplant patients’ microbiota when compared to those of healthy people [4]. Additionally, the same authors reported that patients who developed urinary tract infections had an increase of *Enterococci* [4]. These results were in line with previous studies carried out in bone marrow transplant patients [5].

Therefore, we employed an innovative whole metagenomic profiling approach to find taxonomic, functional and genomic differences of the gut microbiome in a group of renal transplant recipients undergoing maintenance treatment with 2 different immunosuppressive schemas: a less commonly used combined regimen of everolimus (EVE) plus mycophenolate mofetil (MMF) (used in approximately 3% of patients) *versus* a standard immunosuppressive protocol with tacrolimus (TAC) plus MMF.

## Material and methods

### Patients

From February to April 2016 a total of 20 stable adult deceased-donor renal transplant recipients at least 6 months post-transplant [EVE group median (IQR):4.7 years (3.3–6.7) versus TAC group: 5.8 years (4.4–7.4)] were included in this study after signing an informed consent form.

Based on the maintenance immunosuppressive treatment, 9 patients (M/F: 7/2) were treated with everolimus (EVE, Certican, Novartis, levels 3–6 ng/ml) and 11 (M/F: 9/2) with tacrolimus (TAC, Advagraft, Astellas, levels 4–8 ng/ml) in combination with mycophenolate mofetil (MMF, Cell-Cept, Roche) 1000 mg b.i.d. and methylprednisolone 4 mg/day. All patients enrolled received the following induction therapy: 500 mg of methylprednisolone intra-operatively, 250 mg of prednisone daily, with the dose tapered to 25 mg by day 8; 20 mg of a chimeric monoclonal anti-CD25 antibody (Simulect, Novartis) intravenously on day 0 and day 4.

Furthermore, EVE+MMF- and TAC+MMF-treated patients were of similar age (TAC+MMF mean±SD: 60±11.4 *versus* EVE+MMF: 65±7.6 years) and had similar serum creatinine levels [TAC+MMF median (IQR): 107 (95–167) versus EVE+MMF: 99 (87–115) mmol/l] at the time of enrollment.

Patients with biopsy-proven acute rejection in the 6 months prior to the study, as well as those with active infections (including cytomegalovirus (CMV), Epstein-Barr virus (EBV) and BK virus), acute or recurrent urinary tract infections, gastrointestinal disorders, or malignancies at the time of enrollment, were excluded from the study. In addition, patients who received antibiotics, antiviral or nonsteroidal anti-inflammatory drugs during the previous 6 months were not enrolled.

After signing the consent form, patients from both groups were requested to complete a lifestyle and simplified food frequency questionnaire (S1 Table).

The study was carried out according to the principles of the Declaration of Helsinki and was approved by the Verona University Hospital ethics committee (code 752CESC).

Additionally, none of the transplant donors were from a vulnerable population and all donors or next of kin provided written informed consent that was freely given.

### Sample isolation

Nucleic acid isolation was performed with the MoBio PowerMag® Microbiome kit (Carlsbad, CA) according to the manufacturer’s guidelines and optimized for high-throughput processing. All samples were quantified via the Qubit® Quant-iT dsDNA High Sensitivity kit (Invitrogen, NY) to ensure that they met the minimum DNA concentration and quantity requirements.

### Library preparation and raw data processing

Samples were prepared for sequencing with the Illumina Nextera kit and quantified with Quant-iT dsDNA High Sensitivity assays. Libraries were pooled and run with 100-bp paired-end sequencing protocols on the Illumina HiSeq 2500 platform.

Raw sequence reads have been deposited at the European Nucleotide Archive under primary project accession number PRJEB20049.

Host sequences were removed with Kraken [6]. Remaining reads were processed with Trimmomatic [7] to trim adapter sequences and low-quality ends (Q<20). Reads shorter than 35 bp after trimming were discarded. rRNA sequences from all the three domains of life were identified and removed with SortMeRNA 2.0 [8]. Contaminant sequences were removed with Bowtie2 [9].

### Taxonomic profiling

Metagenomic Phylogenetic Analysis, version 2.0 (MetaPhlAn2, [10]) was used for the taxonomic profiling of the metagenomic samples. Raw non-host reads were used directly because low-quality reads were ignored, as were human, 16S rRNA and tRNA reads. Marker genes were identified for bacteria, archaea, viruses and eukaryotic microbes.

### Functional analysis

Filtered DNA sequences were mapped against a reference database of all proteins within the KEGG database (version 75.0). The search for translated DNA sequences was executed using Diamond [11], and hits that spanned ≥20 amino acids with ≥80% similarity were collected.

### Alpha diversity (within-sample diversity) and beta diversity (sample-to-sample dissimilarity) metrics

To evaluate the degree of variation of the microbial community structure within a sample, we measured the alpha diversity by employing the Shannon diversity index [12]. This index utilizes the richness of a sample along with the relative abundance of the detected operational taxonomic units (OTUs, n: 359), functional genes (n: 6520) and pathways (n: 340) to calculate a specific index.

All profiles were compared in a pair-wise fashion to determine a dissimilarity score and were stored in a distance dissimilarity matrix. Abundance-weighted pair-wise differences between samples were calculated using the Bray-Curtis dissimilarity [13]. The binary dissimilarity values were calculated with the Jaccard Index [14].

### Whole-microbiome significance testing

Permutational analysis of variance (PERMANOVA) was utilized to determine significant differences among discrete categorical or continuous variables. PERMANOVA utilizes the sample-to-sample distance matrix directly rather than a derived ordination or clustering outcome.

### Significance testing

Significance testing for OTUs and pathway-level data was accomplished with a Wilcoxon rank sum test.

Univariate differential abundance of functional genes was tested with a negative binomial noise model for the over-dispersion and Poisson process intrinsic to these data, as implemented in the DESeq2 package [15,16]. DESeq was run under default settings, and q-values were calculated with the Benjamini-Hochberg procedure to correct p-values while controlling for false-discovery rates.

## Results

### Taxonomic and pathway and gene functional diversity was similar between the 2 study groups

**Fig 1:**
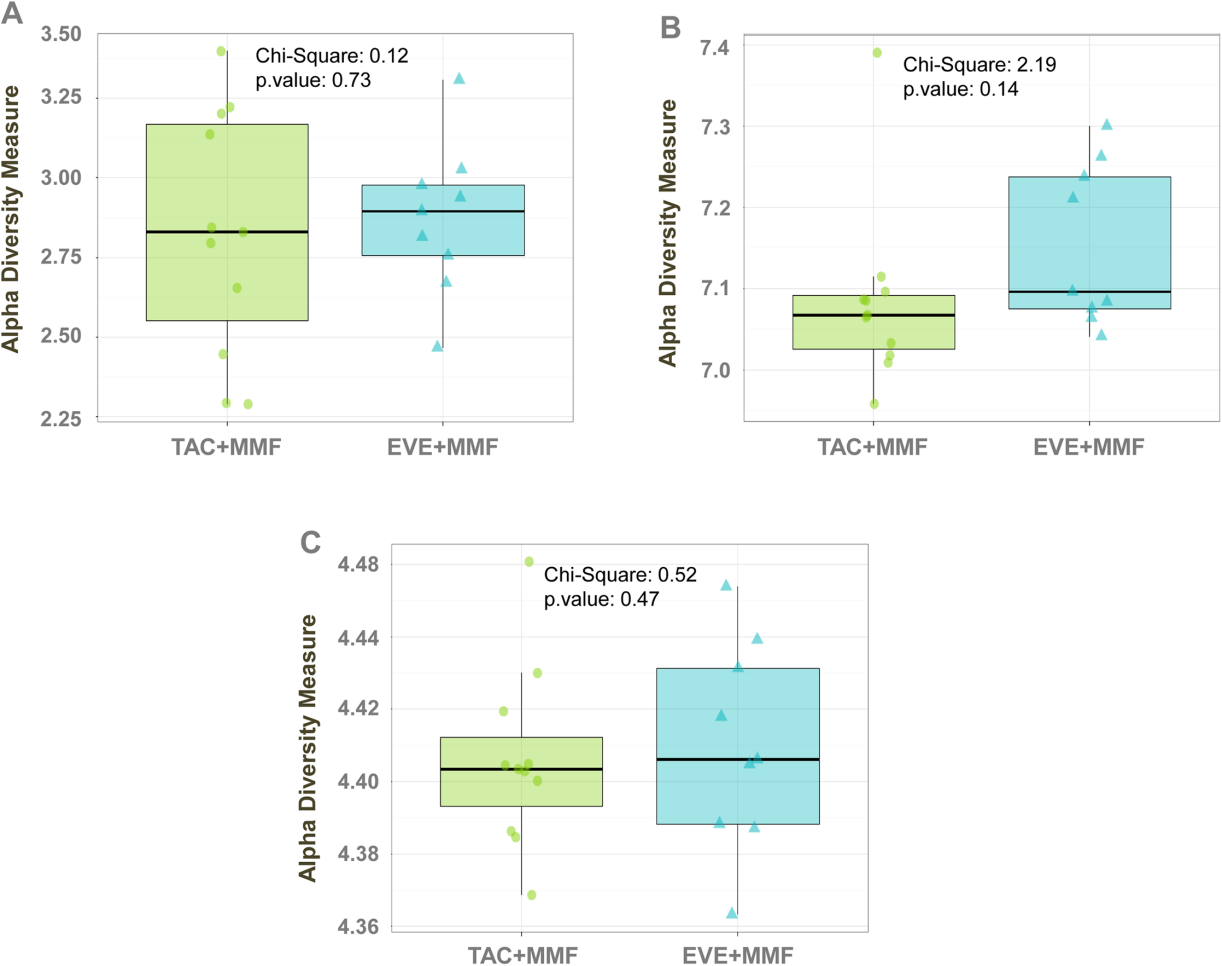
Alpha diversity estimates. Each point shows a sample’s pathway diversity calculated with the Shannon Diversity Index. Samples from both therapies had similar degrees of Shannon diversity at the OTU level (A), microbial gene level (B) and pathway level (C).

As shown in Fig 1, samples from the EVE+MMF group had similar degrees of Shannon diversity at the operational taxonomic unit (OTU) level, microbial gene level and pathway level compared to the TAC+MMF group.

**Fig 2:**
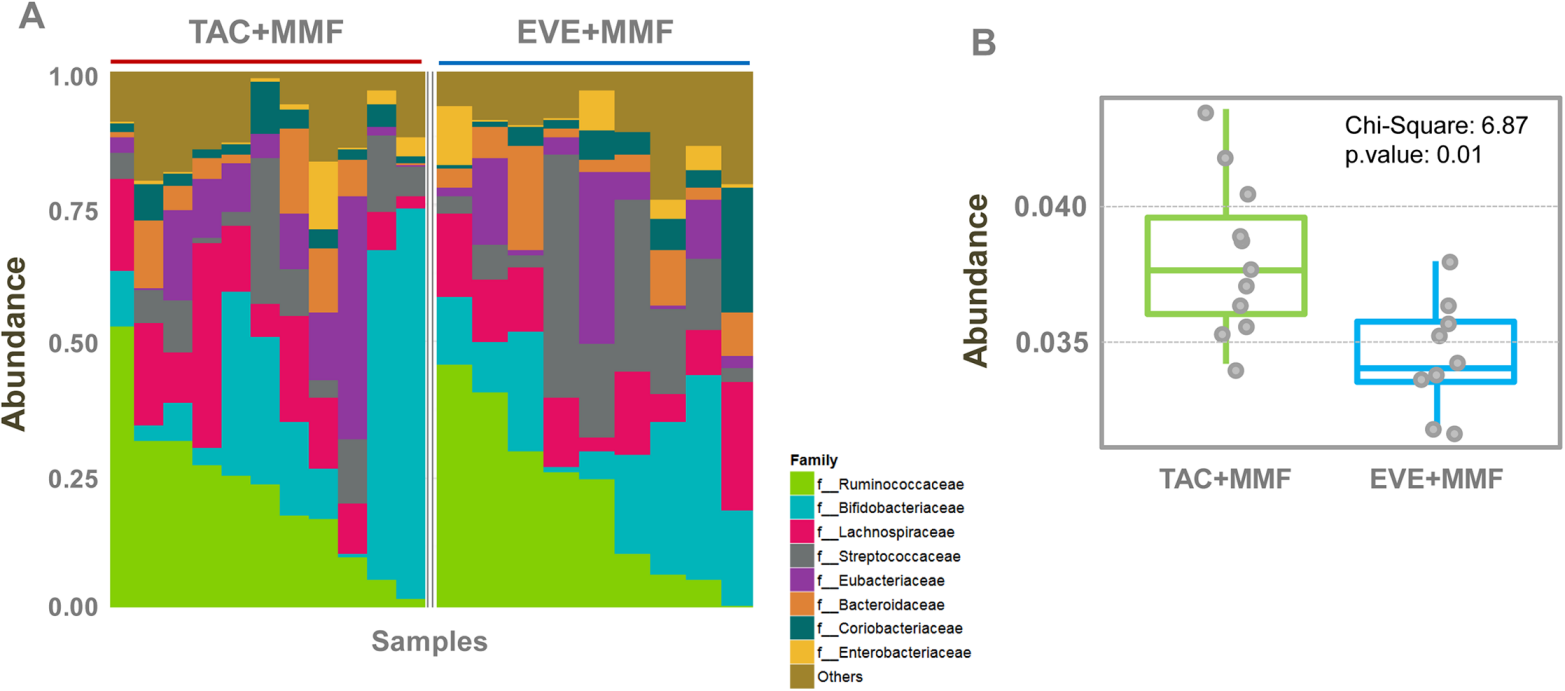
Proportional abundance. (A) The most abundant taxa at the family level. Samples from both therapies had similar abundances of Ruminococcaceae, Bifidobacteriaceae, Lachnospiraceae, Streptococcaceae, Eubacteriaceae, Bacteroidaceae, Coriobacteriaceae and Enterobacteriaceae families. (B) The abundance of starch and sucrose metabolism in the 2 study groups. Compared to the EVE+MMF group, TAC+MMF-treated patients were enriched in this pathway.

Both study groups had a similar abundance of *Ruminococcaceae*, *Bifidobacteriaceae*, *Lachnospiraceae*, *Streptococcaceae*, *Eubacteriaceae*, *Bacteroidaceae*, *Coriobacteriaceae* and *Enterobacteriaceae* families (Fig 2A and Table 1). Interestingly, taxonomic families belonged mainly to phylum Firmicutes (*Ruminococcaceae*, *Lachnospiraceae*, *Streptococcaceae*, *Eubacteriaceae*) which accounted for more than 50% of the gut microbial content, with a relatively low percentage of *Bacteroidetes* (family *Bacteroidaceae*). The different composition compared to the report of Lee et al [4], could also be due to the exclusion of patients exhibiting acute or chronic diarrhea.

**Table 1:** The top 8 most abundant families in the 2 study groups.

Family	Chi-square	KW p-val	TAC+MMF Mean (sd)	EVE+MMF Mean (sd)
Ruminococcaceae	0.0361	0.85	22.5 (14)	21.5 (15.8)
Bifidobacteriaceae	0.0361	0.85	22.1 (24.1)	16.8 (11.5)
Lachnospiraceae	0.1169	0.73	13.7 (9.57)	11.7 (6.14)
Streptococcaceae	0.9019	0.34	8.49 (7.25)	15.1 (14.5)
Eubacteriaceae	0.1746	0.68	10.3 (12.6)	7.98 (10.2)
Bacteroidaceae	0.5209	0.47	5.27 (5.62)	6.22 (5.59)
Coriobacteriaceae	0.1746	0.68	3.43 (2.53)	5.37 (6.86)
Enterobacteriaceae	0.1444	0.70	1.91 (3.57)	2.94 (3.85)

Additionally, in all patients, the highly conserved bacterial ATP-binding cassette, subfamily B (*ABCB-BAC*), putative ATP-binding cassette (*ABC*) transport system permease protein (*ABC*.*CD*.*P*), RNA polymerase beta prime subunit (*rpoC*), RNA polymerase subunit beta (*rpoB*), beta-galactosidase (*lacZ*), periplasmic beta-D-glucoside glucohydrolase (*bgIX*), DNA gyrase subunit A (*gyrA*), and carbamoyl-phosphate synthase large subunit (*carB*, *CPA2*) were the top 8 most abundant functional genes (Table 2 and S1A Fig).

**Table 2:** The top 8 most abundant functional genes in the 2 study groups.

Gene	Chi-square	KW p-val	TAC+MMF Mean (sd)	EVE+MMF Mean (sd)
ABCB-BAC	0.0361	0.85	1.35 (0.149)	1.33 (0.126)
ABC.CD.P	1.7677	0.18	0.768 (0.164)	0.692 (0.0982)
rpoC	1.7677	0.18	0.472 (0.0477)	0.502 (0.0625)
rpoB	1.0519	0.31	0.47 (0.051)	0.493 (0.0531)
lacZ	2.4257	0.12	0.481 (0.0727)	0.42 (0.14)
bglX	3.1876	0.07	0.484 (0.0752)	0.381 (0.128)
gyrA	1.5714	0.21	0.447 (0.0515)	0.411 (0.0522)
carB, CPA2	0.417	0.52	0.43 (0.062)	0.431 (0.071)

Interestingly, although pathway analysis showed no substantial differences between the two study groups (S1B Fig), the samples for renal transplant patients undergoing maintenance treatment with EVE+MMF exhibited a lower abundance of starch and sucrose metabolism pathway genes (Chi-square: 6.87; p-value: 0.01) (S1B Fig and Fig 2B) than those treated with TAC+MMF. This drug-induced microbiomic metabolic change might specifically influence intestinal habit and modify susceptibility to infections.

### Three genes discriminated EVE+MMF from TAC+MMF patients

To identify specific drug-related differences in the microbiome, we employed several bioinformatics algorithms.

**Fig 3:**
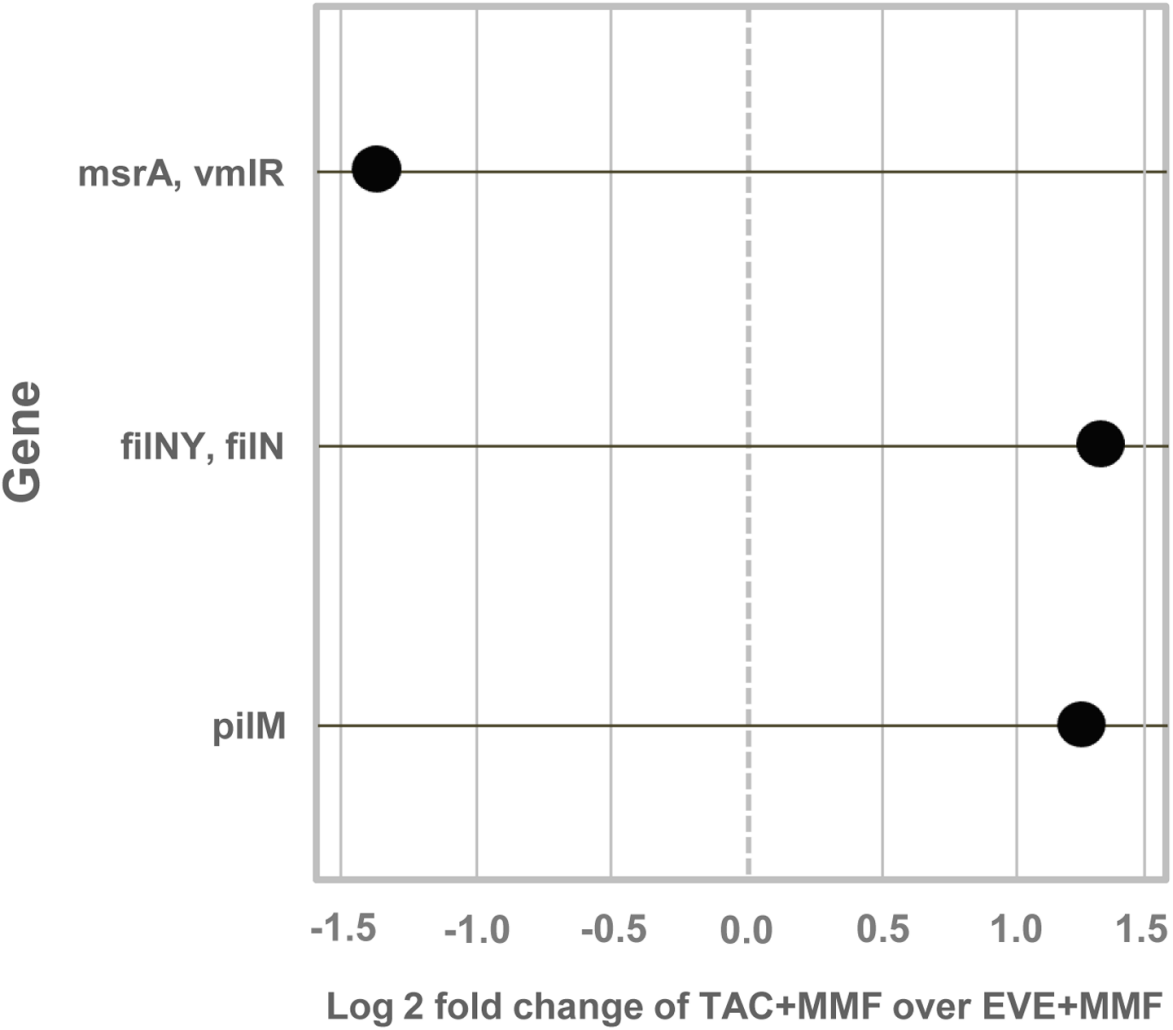
Differentially abundant features. Features were considered significant if their FDR-corrected p-value was less than or equal to 0.05 and the absolute value of the log2 fold change was greater than or equal to 1. Each point represents a functional gene that differentiates TAC+MMF and EVE+MMF therapies. The TAC+MMF group had an enrichment of genes for flagellar motor switch protein (fliNY, fliN) and type IV pilus assembly protein (pilM). EVE+MMF samples had an enrichment of macrolide transport system ATP-binging/permease protein (mrsA, vmlR).

Interestingly, comparative statistical analysis revealed that the 3 top functional genes (detected out of 4515 tested) were able to highly discriminate EVE+MMF *versus* TAC+MMF (cutoff: log2 fold change≥1, FDR≤0.05). Particularly, the macrolide transport system mrsA (*msrA*) was significantly enriched in EVE+MMF, while the flagellar motor switch protein (*fliNY*) and type IV pilus assembly protein pilM (*pilM*) were increased in the TAC+MMF group (Fig 3 and Table 3).

**Table 3:** List of the different functional genes.

KEGG Orthology	Gene	Description	log2 Fold Change	p-value	padj
K02417	fliNY, fliN	flagellar motor switch protein; bacterial chemotaxis and flagellar assembly	1.402666107	3.29E-05	0.04957831
K18231	msrA, vmlR	macrolide transport system ATP-binding/permease protein	-1.451499003	5.31E-06	0.02397658
K02662	pilM	type IV pilus assembly protein PilM (type 2 secretion system)	1.409863436	1.85E-05	0.04185832

In contrast, data analysis revealed that out of the 262 tested, no OTUs passed multiple testing correction. However, 11 OTUs met the log2 fold change threshold and had unadjusted p-values<0.05 (S2 Table). Two of the 11 OTUs were enriched in TAC+MMF-treated subjects, and the rest were enriched in EVE+MMF-treated patients.

One OTU enriched in the TAC+MMF treatment group, *Haemophilus parainfluenzae*, was identified as an opportunistic pathogen. *H*. *parainfluenzae* is often seen in the intestinal microflora [17,18]. However, *H*. *parainfluenzae* OTUs only accounted for a small percentage of the whole microbiomes (mean 0.05% in TAC+MMF and 0.01% in EVE+MMF).

Similarly, no pathways were significantly different between the two study groups after multiple testing correction. Five pathways had an unadjusted p-value<0.05 but did not have an absolute log2 fold change greater than 1 (S3 Table).

### The consumption of sugar was significantly correlated with microbiome composition in renal transplant patients

To estimate the degree of variation of microbial community composition among samples and to better understand which variables significantly contributed to beta diversity, a multivariate analysis that included demographic, clinical and food frequency variables was carried out. Abundance-weighted sample pair-wise differences were calculated using the Bray-Curtis dissimilarity [13].

Notably, PERMANOVA revealed that the consumption of sugar was highly correlated with significant differences in taxonomic (OTU) beta diversity (p-value: 0.0136), functional gene content (p-value: 0.0116), and pathway differences (p-value: 0.0035) among samples (Table 4). The frequency of consumption of sugar is reported in S2 Fig.

**Table 4:** Identification by multivariate (PERMANOVA) analysis of clinical, demographic and food frequency variables significantly contributing to the beta diversity of the samples.

Variable	Class	Taxon p-value	Gene p-value	Path p-value
age	36–77	0.1251	0.474	0.5336
gender	F, M	0.2779	0.6765	0.8047
BMI	20–28.2	0.1069	0.3957	0.3162
TAC level	4–9.2	0.3634	0.7055	0.7408
EVE level	4.8–9.3	0.4741	0.9229	0.4629
comparison	TAC, EVE	0.646	0.626	0.566
creatinine level	65–245	0.4892	0.8314	0.7175
sequences	14442927–27282343	0.59	0.3859	0.1208
sampling timeline	0.52	0.8132	0.57	0.5825
sport hours	0–10	0.158	0.5736	0.5823
sport	No, Yes	0.2059	0.4328	0.2983
barley oats	L0. never, L1. yearly, L2. monthly, L3. weekly, L4. daily	0.1531	0.2826	0.2545
alcoholic drinks	L0. never, L1. yearly, L2. monthly, L3. weekly, L4. daily	0.672	0.4451	0.4939
fish	L0. never, L1. yearly, L2. monthly, L3. weekly	0.1194	0.0889	0.0514
fresh fruit	L0. never, L3. weekly, L4. daily	0.3521	0.0625	0.0559
fresh meat	L0. never, L2. monthly, L3. weekly, L4. daily	0.6368	0.5502	0.5189
legumes	L2. monthly, L3. weekly, L4. daily	0.2832	0.0739	0.0801
nuts	L0. never, L1. yearly, L2. monthly, L3. weekly	0.3652	0.6256	0.506
probiotics	L0. never, L2. monthly, L3. weekly, L4. daily	0.7412	0.662	0.5669
sausages	L0. never, L1. yearly, L2. monthly, L3. weekly, L4. daily	0.5898	0.1888	0.1484
soft drinks	L0. never, L2. monthly, L3. weekly, L4. daily	0.4998	0.747	0.5063
sugar	L0. never, L1. yearly, L2. monthly, L3. weekly, L4. daily	0.0136	0.0116	0.0035
vegetables	L0. never, L4. daily	0.472	0.6275	0.7248
whole grains	L0. never, L1. yearly, L2. monthly, L3. weekly, L4. daily	0.233	0.345	0.4386
gDNA conc	14.553–106.833	0.0663	0.0429	0.0649
MeDi Score	8–13	0.8867	0.6163	0.6903

Interestingly, although it did not reach statistical significance, the consumption of fish, fresh fruit and legumes may have contributed to differences in the microbiome.

**Fig 4:**
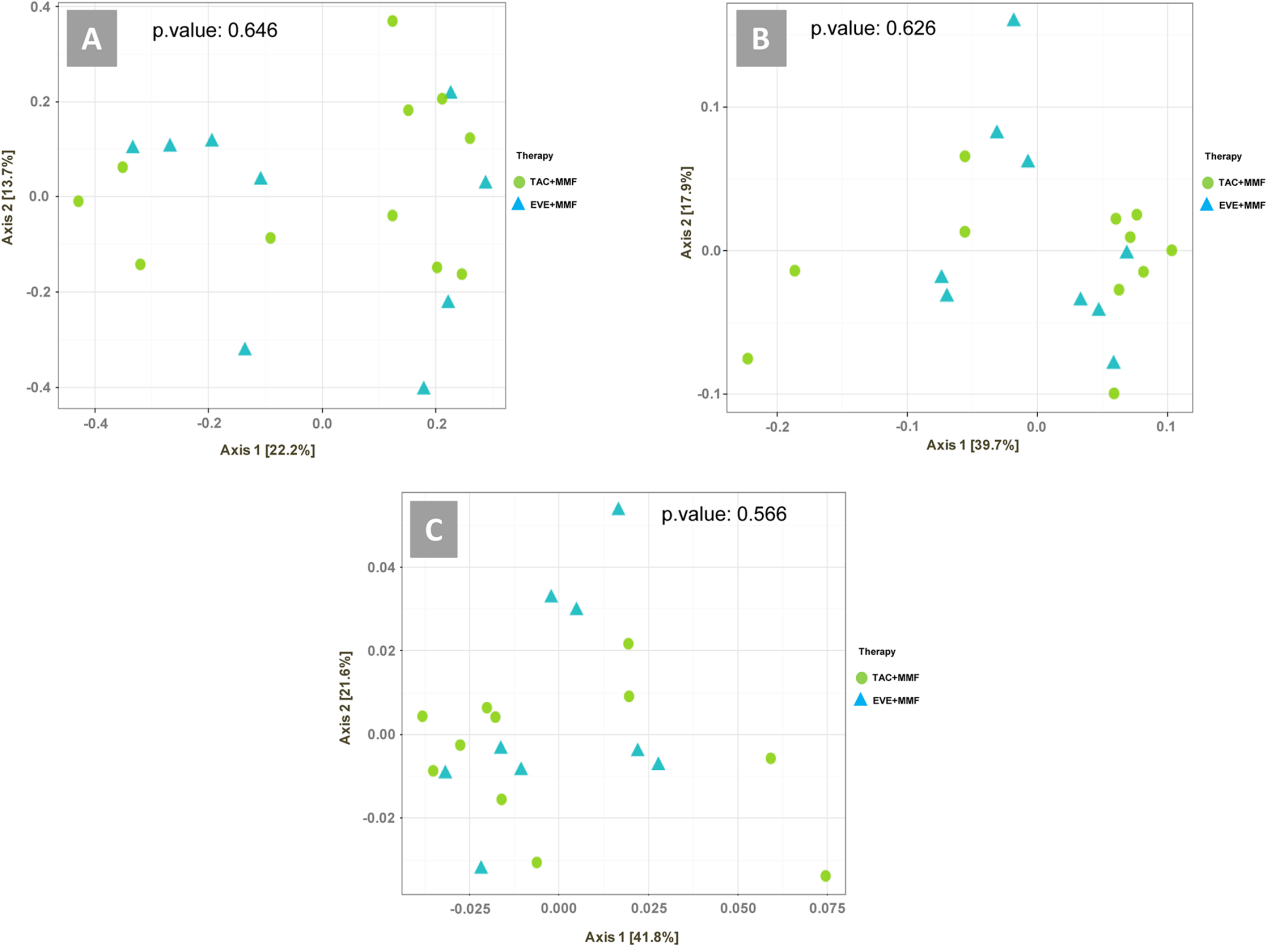
Ordination analysis. Weighted ordination of taxa (A), functional genes (B) and pathway (C) using abundance. Dimensional reduction of the Bray-Curtis distance between microbiome samples using the PCoA ordination method. P-value according to PERMANOVA.

Samples did not cluster or separate according to immunosuppressive therapy in any of the 3 ordination analyses (taxonomy, functional gene, and pathway) (Fig 4).

## Discussion

The relationship between the microbiome and drug response represents a new and fascinating research topic in medicine. However, to date, only a few reports have been published in the field of renal transplantation, none of them are exhaustive [19–21], and none have analyzed the fecal microbiome profile. mTOR-I might be expected to have effects on the microbiome, as mTOR signaling plays a significant role in bacterial recognition by immune cells [22].

In the present study, although performed on a small patient subset (20 patients), we conducted, for the first time, whole fecal metagenomic functional profiling through shotgun sequencing finalized to identify differences between two treatment protocols (EVE+MMF *versus* TAC+MMF). Notably, the small number of patients included was due to 1. the employment of a non-standard immunosuppressive schema based on EVE+MMF (used in approximately 3% of patients); 2. the strict inclusion criteria finalized to minimize confounding demographic, clinical, environmental and pharmacological variables; and 3. the high cost of the methodology used.

The methodology utilized in our study allowed us to comprehensively analyze a large number of genes in all organisms present in fecal samples, enabling us to evaluate bacterial diversity and to detect the abundance of microbes. This method of sequencing can detect very low-abundance members of the microbial community that may be missed using other methodologies. However, because of the large amount of metagenomic data and the presence of specific technical problems, skilled bioinformaticians, in addition to novel and efficient computational tools, are required [23].

Interestingly, our statistical analysis demonstrated that although the two groups of patients exhibited a similar degree of alpha diversity at the taxonomic and gene/pathway expression level, a comparative high-throughput analysis revealed that three functional genes clearly discriminated EVE+MMF *versus* TAC+MMF (cutoff: log2-fold change≥1, FDR≤0.05). The flagellar motor switch protein (*fliNY*) and type IV pilus assembly protein pilM (*pilM*) were significantly enriched in TAC+MMF-treated patients, while the macrolide transport system mrsA (*msrA*) was increased in patients treated with EVE+MMF.

The bacterial flagellum is a component shared by several pathogenic species, including S*pirochetes*, *E*. *coli* [24] and *Salmonella*. This filamentous organelle mediates the movement in liquid or semi-solid environments and chemotaxis. The flagellum components, in particular the protein encoded by the *FliN* gene, give a clockwise or counterclockwise motion to direct bacterial movement. It has also been observed that the deletion of structural proteins of the flagellum can lead to a reduction in bacterial virulence [25,26].

*Bacterial pili* are hair-like structures on the cell surface of many bacteria that mediate the adherence to surfaces or eukaryotic cells. PilM is a cytoplasmic actin-like protein that binds to the short cytoplasmic N-terminus of the inner membrane protein PilN and, together with inner membrane proteins PilO and PilNO and lipoproteins PilP and PilQ, constitutes the secretin alignment subcomplex [27–29] of type IV pili. The PilMNOP complex is required for efficient pilus assembly [30–32]. Type IV pili have several functions, including gliding motility, protein secretion, adherence to eukaryotic cells, and twitching motility [33], which play a key role in the rapid colonization of new surfaces under conditions of high nutrient availability and in the development of biofilm [34].

A biofilm is an assemblage of different species of microbial cells irreversibly associated with a surface and enclosed in a matrix of primarily polysaccharide material [35]. Interestingly, this complex system also has a role in antimicrobial resistance through several mechanisms: cells may exchange resistance plasmids within biofilms, bacteria in biofilms are less susceptible to antibiotics, biofilm-associated gram-negative bacteria may produce endotoxins, and biofilms are resistant to host immune system clearance [35].

Recent data have demonstrated that type IV pili can also sense mechanical features of their environment and regulate surface-induced gene expression and pathogenicity [36]. In fact, type IV assembly systems are functionally related to the type II secretion system, which is responsible for the extrusion of folded proteins, including proteases, cellulases, pectinases, phospholipases, lipases, and toxins contributing to cell damage and disease [37,38] and bacterial survival.

The *msr*(A) gene encodes an ABC transporter protein that constitutes an efflux pump mediating the bacterial resistance to macrolide [39].

Multi-drug efflux pumps (MEFs) are membrane protein complexes that allow the extrusion of various substrates from the cells, particularly many antibiotics; they represent a well-encoded bacterial resistance mechanism shared by numerous species, e.g., Enterobacteriaceae [40], which are responsible for sepsis and urinary tract infections in transplant patients. This is a molecular target of scientific research to create new antimicrobial drugs [23,41].

Moreover, the expression of the *bacterial pilus*, *flagellar motor protein* and MEF pumps are characteristics of the *Enterobacteriaceae* group, which include *Klebsiella pneumoniae*, one of the main pathogens responsible for urinary tract infections (UTIs) [42].

Recurrent UTIs, often caused by multi-drug-resistant organisms, are among the most frequent infectious diseases in the kidney transplant population. Although they are not associated with an increased mortality or organ loss, they often require prolonged hospitalization and intravenous antibiotic therapies, with higher costs and risks for the patient [43,44].

Interestingly, an analysis of the available literature showed that the overall taxonomic and genetic composition of the microbiomes of our renal transplant recipients was similar to that of healthy subjects [45]. This result was probably due to the plasticity of the gut microbiota and the absence of a significant renal functional impairment in our patients.

Additionally, our analysis revealed that the consumption of sugar was highly correlated with significant differences in taxonomic (OTU) beta diversity (p-value: 0.0136), functional gene content (p-value: 0.0116), and pathway differences (p-value: 0.0035) among samples. This finding demonstrated that compared to immunosuppressive drugs, diet likely had a major impact on variation in the gut microbiota composition.

Among all considered food categories, sugar significantly influenced beta diversity. For a long time, the Western diet, which is high in sugars and fats, has been associated with dysbiosis, obesity, diabetes and metabolic syndrome [46–49]. Our findings confirmed that diet is a factor capable, in a small period of time [50], of exerting selective pressure on microbial species and on microbial genes and pathways that are functionally expressed in the intestinal microbial community. On the other hand, considering taxonomic composition, the taxonomic families allotted to phylum Firmicutes (*Ruminococcaceae*, *Lachnospiraceae*, *Streptococcaceae*, *Eubacteriaceae*) constituted, on average, the majority of microbial taxa present in the gut, while the other most important phylum Bacteroidetes seemed to be poorly represented. This is in line with observations reported by Lee and colleagues, who reported that Firmicutes and Bacteroidetes in post-transplantation fecal specimens, were, respectively, the most abundant bacterial taxa and much lower than observed in samples from healthy subjects analyzed by the Human Microbiome Consortium [4]. The same observation is valid when considering the relative prevalence of Firmicutes and Bacteroidetes in stool samples from healthy Italian donors [51].

In conclusion, our study, although performed on a limited sample size, showed, for the first time, immunosuppressive-related effects on the fecal microbiome although the enrichment of genes we found may have resulted from indirect effects and, while they may serve as useful biomarkers, may not directly guide therapeutic interventions.

However we cannot exclude that in future a larger employment of metagenomics, could lead to a breakthrough in understanding the effects of immunosuppressive drugs routinely employed in renal and other solid organs transplantation. Undoubtedly, because results of this study are mainly obtained analyzing a restricted number of variables correlated with the whole gut microbial metagenomic data, they cannot be considered definitive. Multicenter studies, including more clinical/pharmacological variables and a large number of patients and healthy controls should be undertaken to obtain definitive results.

## Supporting information

S1 Fig Most abundant functional genes (A) and pathways (B) in the two groups of patients.
(DOCX)

S2 Fig Percentage distribution of sugar consumption in the samples. (DOCX)

S1 Table Life style and simplified food frequency questionnaire. (DOCX)

S2 Table List of the OTUs that had an unadjusted p-values <0.05 and absolute log 2 fold change>1. (DOCX)

S3 Table List of pathways that had an unadjusted p-value<0.05 but did not meet the log 2 fold change threshold. (DOCX)

## References

[1] D'Argenio V, Salvatore F. The role of the gut microbiome in the healthy adult status. Clin Chim Acta. 2015; 451(Pt A): 97–102 doi: 10.1016/j.cca.2015.01.003 25584460 10.1016/j.cca.2015.01.003

[2] Wilson ID, Nicholson JK. Gut microbiome interactions with drug metabolism, efficacy, and toxicity. Transl Res. 2017; 179: 204–222 doi: 10.1016/j.trsl.2016.08.002 27591027 10.1016/j.trsl.2016.08.002 PMC5718288

[3] Lee JR, Muthukumar T, Dadhania D, Taur Y, Jenq RR, Toussaint NC, et al Gut microbiota and tacrolimus dosing in kidney transplantation. PLoS One. 2015; 10(3): e0122399 doi: 10.1371/journal.pone.0122399 25815766 10.1371/journal.pone.0122399 PMC4376942

[4] Lee JR, Muthukumar T, Dadhania D, Toussaint NC, Ling L, Pamer E, et al Gut microbial community structure and complications after kidney transplantation: a pilot study. Transplantation. 2014; 98(7): 697–705. doi: 10.1097/TP.0000000000000370 25289916 10.1097/TP.0000000000000370 PMC4189837

[5] Ubeda C, Taur Y, Jenq RR, Equinda MJ, Son T, Samstein M, et al Vancomycin-resistant Enterococcus domination of intestinal microbiota is enabled by antibiotic treatment in mice and precedes bloodstream invasion in humans. J Clin Invest. 2010; 120(12): 4332–4341 doi: 10.1172/JCI43918 21099116 10.1172/JCI43918 PMC2993598

[6] Wood DE, Salzberg SL. Kraken: ultrafast metagenomic sequence classification using exact alignments. Genome Biol. 2014; 15(3): R46 doi: 10.1186/gb-2014-15-3-r46 24580807 10.1186/gb-2014-15-3-r46 PMC4053813

[7] Kopylova E, Noé L, Touzet H. SortMeRNA: fast and accurate filtering of ribosomal RNAs in metatranscriptomic data. Bioinformatics. 2012; 28(24): 3211–3217. doi: 10.1093/bioinformatics/bts611 23071270 10.1093/bioinformatics/bts611

[8] Bolger AM, Lohse M, Usadel B. Trimmomatic: a flexible trimmer for Illumina sequence data. Bioinformatics. 2014; 30(15): 2114–2120. doi: 10.1093/bioinformatics/btu170 24695404 10.1093/bioinformatics/btu170 PMC4103590

[9] Langmead B, Salzberg SL. Fast gapped-read alignment with Bowtie 2. Nat Methods. 2012; 9(4): 357–359 doi: 10.1038/nmeth.1923 22388286 10.1038/nmeth.1923 PMC3322381

[10] Truong DT, Franzosa EA, Tickle TL, Scholz M, Weingart G, Pasolli E, et al MetaPhlAn2 for enhanced metagenomic taxonomic profiling. Nat Methods. 2015; 12(10): 902–903. doi: 10.1038/nmeth.3589 26418763 10.1038/nmeth.3589

[11] Buchfink B, Xie C, Huson DH. Fast and sensitive protein alignment using DIAMOND. Nat Methods. 2015; 12(1): 59–60 doi: 10.1038/nmeth.3176 25402007 10.1038/nmeth.3176

[12] Shannon’s Diversity Index: a mathematical theory of communication. Shannon C.E. The Bell System Technical Journal (1948) 27, 379–423 and 623–656

[13] Bray JR, Curtis JT. An ordination of upland forest communities of southern Wisconsin. Ecological Monographs 1957; 27:325–349.

[14] Jaccard P. The distribution of the flora in the alpine zone. The new Phytologist 1912; 11(2): 37–50

[15] Love MI, Huber W, Anders S. Moderated estimation of fold change and dispersion for RNA-seq data with DESeq2. Genome Biol. 2014; 15(12): 550 doi: 10.1186/s13059-014-0550-8 25516281 10.1186/s13059-014-0550-8 PMC4302049

[16] McMurdie PJ, Holmes S. phyloseq: an R package for reproducible interactive analysis and graphics of microbiome census data. PLoS One. 2013; 8(4): e61217 doi: 10.1371/journal.pone.0061217 23630581 10.1371/journal.pone.0061217 PMC3632530

[17] Martel AY, St-Laurent G, Dansereau LA, Bergeron MG. Isolation and biochemical characterization of Haemophilus species isolated simultaneously from the oropharyngeal and anogenital areas. J Clin Microbiol. 1989; 27(7):1486–1489. 2671014 10.1128/jcm.27.7.1486-1489.1989 PMC267600

[18] Kasai C, Sugimoto K, Moritani I, Tanaka J, Oya Y, Inoue H, et al Comparison of human gut microbiota in control subjects and patients with colorectal carcinoma in adenoma: Terminal restriction fragment length polymorphism and next-generation sequencing analyses. Oncol Rep. 2016; 35(1):325–333 doi: 10.3892/or.2015.4398 26549775 10.3892/or.2015.4398

[19] Fricke WF, Maddox C, Song Y, Bromberg JS. Human microbiota characterization in the course of renal transplantation. Am J Transplant. 2014; 14(2):416–427 doi: 10.1111/ajt.12588 24373208 10.1111/ajt.12588

[20] Rani A, Ranjan R, McGee HS, Andropolis KE, Panchal DV, Hajjiri Z, et al Urinary microbiome of kidney transplant patients reveals dysbiosis with potential for antibiotic resistance. Transl Res. 2017; 181:59–70. doi: 10.1016/j.trsl.2016.08.008 27669488 10.1016/j.trsl.2016.08.008 PMC5344767

[21] Rani A, Ranjan R, McGee HS, Metwally A, Hajjiri Z, Brennan DC, et al A diverse virome in kidney transplant patients contains multiple viral subtypes with distinct polymorphisms. Sci Rep. 2016 9 16;6:33327 doi: 10.1038/srep33327 27633952 10.1038/srep33327 PMC5025891

[22] Abdel-Nour M, Tsalikis J, Kleinman D, Girardin SE. The emerging role of mTOR signalling in antibacterial immunity. Immunol Cell Biol. 2014; 92(4):346–353. doi: 10.1038/icb.2014.3 24518980 10.1038/icb.2014.3

[23] Mulcahy-O'Grady H, Workentine ML. The Challenge and Potential of Metagenomics in the Clinic. Front Immunol. 2016; 7:29 doi: 10.3389/fimmu.2016.00029 26870044 10.3389/fimmu.2016.00029 PMC4737888

[24] Li C, Motaleb A, Sal M, Goldstein SF, Charon NW. Spirochete periplasmic flagella and motility. J Mol Microbiol Biotechnol. 2000; 2(4): 345–354. 11075905

[25] Li C, Xu H, Zhang K, Liang FT. Inactivation of a putative flagellar motor switch protein FliG1 prevents Borrelia burgdorferi from swimming in highly viscous media and blocks its infectivity. Mol Microbiol. 2010; 75(6): 1563–1576 doi: 10.1111/j.1365-2958.2010.07078.x 20180908 10.1111/j.1365-2958.2010.07078.x PMC4394363

[26] Liao S, Sun A, Ojcius DM, Wu S, Zhao J, Yan J. Inactivation of the FliY gene encoding a flagellar motor switch protein attenuates mobility and virulence of Leptospira interrogans strain Lai. BMC Microbiol. 2009; 9:253 doi: 10.1186/1471-2180-9-253 20003186 10.1186/1471-2180-9-253 PMC3224694

[27] Sampaleanu LM, Bonanno JB, Ayers M, Koo J, Tammam S, Burley SK, et al Periplasmic domains of Pseudomonas aeruginosa PilN and PilO form a stable heterodimeric complex. J Mol Biol. 2009; 394(1): 143–159 doi: 10.1016/j.jmb.2009.09.037 19857646 10.1016/j.jmb.2009.09.037

[28] Karuppiah V, Derrick JP. Structure of the PilM-PilN inner membrane type IV pilus biogenesis complex from Thermus thermophilus. J Biol Chem. 2011; 286(27): 24434–24442 doi: 10.1074/jbc.M111.243535 21596754 10.1074/jbc.M111.243535 PMC3129222

[29] Tammam S, Sampaleanu LM, Koo J, Sundaram P, Ayers M, Chong PA, et al Characterization of the PilN, PilO, and PilP type IVa pilus subcomplex. Mol Microbiol. 2011; 82(6): 1496–1514 doi: 10.1111/j.1365-2958.2011.07903.x 22053789 10.1111/j.1365-2958.2011.07903.x

[30] Ayers M, Sampaleanu LM, Tammam S, Koo J, Harvey H, Howell PL, et al PilM/N/O/P proteins form an inner membrane complex that affects the stability of the Pseudomonas aeruginosa type IV pilus secretin. J Mol Biol. 2009; 394(1): 128–142. doi: 10.1016/j.jmb.2009.09.034 19857645 10.1016/j.jmb.2009.09.034

[31] Carbonnelle E, Helaine S, Nassif X, Pelicic V. A systematic genetic analysis in Neisseria meningitidis defines the Pil proteins required for assembly, functionality, stabilization and export of type IV pili. Mol Microbiol. 2006; 61(6): 1510–1522. doi: 10.1111/j.1365-2958.2006.05341.x 16968224 10.1111/j.1365-2958.2006.05341.x

[32] Nudleman E, Wall D, Kaiser D. Polar assembly of the type IV pilus secretin in Myxococcus xanthus. Mol Microbiol. 2006; 60(1): 16–29. doi: 10.1111/j.1365-2958.2006.05095.x 16556217 10.1111/j.1365-2958.2006.05095.x

[33] Melville S, Craig L. Type IV pili in Gram-positive bacteria. Microbiol Mol Biol Rev. 2013; 77(3): 323–341 doi: 10.1128/MMBR.00063-12 24006467 10.1128/MMBR.00063-12 PMC3811610

[34] Mattick JS. Type IV pili and twitching motility. Annu Rev Microbiol. 2002; 56: 289–314. doi: 10.1146/annurev.micro.56.012302.160938 12142488 10.1146/annurev.micro.56.012302.160938

[35] Donlan RM. Biofilms: microbial life on surfaces. Emerg Infect Dis. 2002; 8(9): 881–890 doi: 10.3201/eid0809.020063 12194761 10.3201/eid0809.020063 PMC2732559

[36] Persat A, Inclan YF, Engel JN, Stone HA, Gitai Z. Type IV pili mechanochemically regulate virulence factors in Pseudomonas aeruginosa. Proc Natl Acad Sci U S A. 2015; 112(24): 7563–7568 doi: 10.1073/pnas.1502025112 26041805 10.1073/pnas.1502025112 PMC4475988

[37] Sandkvist M. Type II secretion and pathogenesis. Infect Immun. 2001; 69(6): 3523–3535 doi: 10.1128/IAI.69.6.3523-3535.2001 11349009 10.1128/IAI.69.6.3523-3535.2001 PMC98326

[38] Ayers M, Howell PL, Burrows LL. Architecture of the type II secretion and type IV pilus machineries. Future Microbiol. 2010; 5(8): 1203–1218 doi: 10.2217/fmb.10.76 20722599 10.2217/fmb.10.76

[39] Ross JI, Eady EA, Cove JH, Cunliffe WJ, Baumberg S, Wootton JC. Inducible erythromycin resistance in staphylococci is encoded by a member of the ATP-binding transport super-gene family. Mol Microbiol. 1990; 4(7): 1207–1214. 2233255 10.1111/j.1365-2958.1990.tb00696.x

[40] Andersen JL, He GX, Kakarla P, K C R, Kumar S, Lakra WS, et al Multidrug efflux pumps from Enterobacteriaceae, Vibrio cholerae and Staphylococcus aureus bacterial food pathogens. Int J Environ Res Public Health. 2015; 12(2): 1487–1547. doi: 10.3390/ijerph120201487 25635914 10.3390/ijerph120201487 PMC4344678

[41] Schmieder R, Edwards R. Insights into antibiotic resistance through metagenomic approaches. Future Microbiol. 2012; 7(1):73–89 doi: 10.2217/fmb.11.135 22191448 10.2217/fmb.11.135

[42] Silva C, Afonso N, Macário F, Alves R, Mota A. Recurrent urinary tract infections in kidney transplant recipients. Transplant Proc. 2013;45(3):1092–5 doi: 10.1016/j.transproceed.2013.02.019 23622634 10.1016/j.transproceed.2013.02.019

[43] Mitra S, Alangaden GJ. Recurrent urinary tract infections in kidney transplant recipients. Curr Infect Dis Rep. 2011; 13(6): 579–587 doi: 10.1007/s11908-011-0210-z 21870039 10.1007/s11908-011-0210-z

[44] Bodro M, Sanclemente G, Lipperheide I, Allali M, Marco F, Bosch J, et al Impact of antibiotic resistance on the development of recurrent and relapsing symptomatic urinary tract infection in kidney recipients. Am J Transplant. 2015; 15(4): 1021–1027. doi: 10.1111/ajt.13075 25676738 10.1111/ajt.13075

[45] Human Microbiome Consortium. Structure, function and diversity of the healthy human microbiome. Nature. 2012; 486(7402): 207–214 doi: 10.1038/nature11234 22699609 10.1038/nature11234 PMC3564958

[46] Turnbaugh PJ, Bäckhed F, Fulton L, Gordon JI. Diet-induced obesity is linked to marked but reversible alterations in the mouse distal gut microbiome. Cell Host Microbe. 2008; 3(4): 213–223 doi: 10.1016/j.chom.2008.02.015 18407065 10.1016/j.chom.2008.02.015 PMC3687783

[47] Zhao L. The gut microbiota and obesity: from correlation to causality. Nat Rev Microbiol. 2013; 11: 639–647 doi: 10.1038/nrmicro3089 23912213 10.1038/nrmicro3089

[48] Turnbaugh PJ, Ley RE, Mahowald MA, Magrini V, Mardis ER, Gordon JI. An obesity-associated gut microbiome with increased capacity for energy harvest. Nature. 2006; 444(7122): 1027–1031. doi: 10.1038/nature05414 17183312 10.1038/nature05414

[49] Karlsson F, Tremaroli V, Nielsen J, Bäckhed F. Assessing the human gut microbiota in metabolic diseases. Diabetes. 2013; 62(10):3341–3349 doi: 10.2337/db13-0844 24065795 10.2337/db13-0844 PMC3781439

[50] David LA, Maurice CF, Carmody RN, Gootenberg DB, Button JE, Wolfe BE, et al Diet rapidly and reproducibly alters the human gut microbiome. Nature. 2014; 505(7484): 559–563. doi: 10.1038/nature12820 24336217 10.1038/nature12820 PMC3957428

[51] De Filippis F, Pellegrini N, Vannini L, Jeffery IB, La Storia A, Laghi L, et al High-level adherence to a Mediterranean diet beneficially impacts the gut microbiota and associated metabolome. Gut. 2016; 65(11):1812–1821 doi: 10.1136/gutjnl-2015-309957 26416813 10.1136/gutjnl-2015-309957

