# Supplementary material for: Impact of maintenance immunosuppressive therapy on the fecal microbiome of renal transplant recipients: Comparison between an everolimus- and a standard tacrolimus-based regimen
Source: PLoS One. 2017 May 24;12(5):e0178228. doi: 10.1371/journal.pone.0178228 (PMC5443527; doi:10.1371/journal.pone.0178228)
Supplement: S1 Fig — Most abundant functional genes (A) and pathways (B) in the two groups of patients. (DOCX) [file pone.0178228.s001.docx]

**S1 Fig.** Most abundant functional genes (A) and pathways (B) in the two groups of patients.
