# Supplementary material for: Impact of maintenance immunosuppressive therapy on the fecal microbiome of renal transplant recipients: Comparison between an everolimus- and a standard tacrolimus-based regimen
Source: PLoS One. 2017 May 24;12(5):e0178228. doi: 10.1371/journal.pone.0178228 (PMC5443527; doi:10.1371/journal.pone.0178228)
Supplement: S1 Table — (DOCX) [file pone.0178228.s003.docx]

**S1 Table.** Life style and simplified food frequency questionnaire

**General data**

**Name:** …………………..……………… **Surname:** ………………………………………………

**Age:** *..........*

**Gender** M F

**Smoking habit** No Yes

**Weight** (Kg) *..........*

**Height** (cm) *..........*

**Food frequency**

**Do you follow a special diet?** No Yes *(If yes, please specify)*

(E.g. vegetarian, zone diet, etc.)

*.......………………………………*

**Do you have celiac disease?** No Yes

**Do you have food allergy** No Yes *(If yes, please specify)*

**and food intolerance?**  *....……………………………………*

**Do you dietary supplements?**  No Yes

**If yes, how often?**  Occasionally Regularly

**If yes, which dietary supplements do you use?**

**(you can tick more than one box)** Vitamins

Minerals

Vitamins and minerals

Proteins/aminoacids

Fibers (If yes, please specify)……………………

Antioxidants

Probiotics (lactic ferments, yogurt) If yes, please specify the brand……………….……………………

**Do you practice sports or physical exercise?** Yes No *If yes please specify?*………………

How often? Daily 1-3 times a week Seldom Total hours per week ……………..

**Frequency of consumption of food groups**

(Tick one box on each line)

| **How often do you eat the following foods?** | Never | Day  (1-3 times) | Week  (1-3 times) | Month  (1-3 times) | Year  (Seldom) |
| --- | --- | --- | --- | --- | --- |
| **Cereals and refined grains** *(E.g.: pasta, rice, bread, pizza, crackers, biscuits, cornflakes, etc.)* |  |  |  |  |  |
| **Whole grains foods** *(E.g.: pasta, bread, crackers, biscuits, rusks, etc.)* |  |  |  |  |  |
| **Barley and oat**  *(beans or flakes)* |  |  |  |  |  |
| **Fresh meats** |  |  |  |  |  |
| **Processed meats**  (*E.g.: ham, salami, wurstel, etc*.) |  |  |  |  |  |
| **Fish and seafood**  *(E.g.: bass, octopus, shrimp, etc.)* |  |  |  |  |  |
| **Milk and dairy products**  *(E.g.: fresh and seasoned cheeses)* |  |  |  |  |  |
| **Yogurt and probiotic foods** |  |  |  |  |  |
| **Fresh fruits** |  |  |  |  |  |
| **Dried fruits**  *(E.g.: walnuts, hazelnuts, almonds, etc.)* |  |  |  |  |  |
| **Vegetables** *(* *Ex: lettuce, chicory, endive, spinach, beets, etc. )* |  |  |  |  |  |
| **Legumes**  *(E.g.: beans, lentils, peas, chickpeas, etc.)* |  |  |  |  |  |
| **Eggs** |  |  |  |  |  |
| **Sweets**  *(E.g.: cakes, pies, ice-creams, chocolates, etc.)* |  |  |  |  |  |
| **Soft drinks**  *(E.g.: Coke, soda, orange juice, etc. )* |  |  |  |  |  |
| **Alcoholic drinks**  *(E.g.: wine, beer, spirits, etc.)* |  |  |  |  |  |
