# Supplementary material for: Impact of maintenance immunosuppressive therapy on the fecal microbiome of renal transplant recipients: Comparison between an everolimus- and a standard tacrolimus-based regimen
Source: PLoS One. 2017 May 24;12(5):e0178228. doi: 10.1371/journal.pone.0178228 (PMC5443527; doi:10.1371/journal.pone.0178228)
Supplement: S2 Table — (DOCX) [file pone.0178228.s004.docx]

**S2 Table.** List of the OTUs that had an unadjusted p-values <0.05 and absolute log 2 fold change>1.

Multiple testing correction (padj) method was FDR
