# Supplementary material for: Impact of maintenance immunosuppressive therapy on the fecal microbiome of renal transplant recipients: Comparison between an everolimus- and a standard tacrolimus-based regimen
Source: PLoS One. 2017 May 24;12(5):e0178228. doi: 10.1371/journal.pone.0178228 (PMC5443527; doi:10.1371/journal.pone.0178228)
Supplement: S3 Table — (DOCX) [file pone.0178228.s005.docx]

**S3 Table.** List of pathways that had an unadjusted p-value<0.05 but did not meet the log 2 fold change threshold.

Multiple testing correction (padj) method was FDR
